# Supplementary material for: Evaluation of the Climate Impact and Nutritional Quality of Menus in an Italian Long-Term Care Facility
Source: Nutrients. 2024 Aug 23;16(17):2815. doi: 10.3390/nu16172815 (PMC11396820; doi:10.3390/nu16172815)
Supplement: Supplementary file 1 [file nutrients-16-02815-s001.zip › nutrients-3160112-supplementary.pdf]

# Supplementary material

Table S1: **Cereals, cereals products, and substitutes**

| Food item | CF (g $CO_2$/g) | White meat (%) | Red meat (%) | Cereals (%) | Sweets (%) | Fruit (%) | Fats (%) | Milk (%) | Cheese (%) | Legumes (%) | Olive oil (%) | Fish (%) | Vegetables (%) | Potatoes (%) | Eggs (%) | Sunflower oil (%) |
|---|---|---|---|---|---|---|---|---|---|---|---|---|---|---|---|---|
| Barley[1] | 0.71 | | | 100 | | | | | | | | | | | | |
| Bread[1] | 1 | | | 100 | | | | | | | | | | | | |
| Breadcrumbs[1] | 1 | | | 100 | | | | | | | | | | | | |
| Corn[1] | 0.45 | | | 100 | | | | | | | | | | | | |
| Corn flour[1] | 0.45 | | | 100 | | | | | | | | | | | | |
| Croutons[1] | 1.46 | | | 100 | | | | | | | | | | | | |
| Gnocchi alla romana[2, A] | 1.6 | | | 18 | | | 2 | 70 | 6 | | | | | | 4 | |
| Lasagna pasta[1] | 1.1 | | | 80 | | | | | | | | | | | 20 | |
| Meat ravioli[1] | 1.1 | | 30 | 55 | | | | | | | | | | | 15 | |
| Meat tortellini[1] | 1.1 | | 30 | 55 | | | | | | | | | | | 15 | |
| Potato gnocchi[1] | 0.375 | | | 27 | | | | | | | | | | 73 | | |
| Pasta for broth[1] | 1.07 | | | 100 | | | | | | | | | | | | |
| Pizza margherita[1] | 1.3 | | | 59 | | | | | 20 | | | | 20 | | | 1 |
| Puff pastry[1, B] | 1.53 | | | 46 | | | 36 | | | | | | | | | |
| Rice[1] | 2.76 | | | 100 | | | | | | | | | | | | |
| Rusks[1] | 1.46 | | | 100 | | | | | | | | | | | | |
| Semolina pasta[1] | 1.07 | | | 100 | | | | | | | | | | | | |
| Spelt[1] | 0.57 | | | 100 | | | | | | | | | | | | |
| Vegetarian ravioli[1] | 1.1 | | | 31 | | | | | 21 | | | | 23 | | 25 | |
| Wheat flour[1] | 0.71 | | | 100 | | | | | | | | | | | | |
| Wheat semolina[1] | 0.71 | | | 100 | | | | | | | | | | | | |

Table S2: **Legumes**

| Food item | CF (g $CO_2$/g) | White meat (%) | Red meat (%) | Cereals (%) | Sweets (%) | Fruit (%) | Fats (%) | Milk (%) | Cheese (%) | Legumes (%) | Olive oil (%) | Fish (%) | Vegetables (%) | Potatoes (%) | Eggs (%) | Sunflower oil (%) |
|---|---|---|---|---|---|---|---|---|---|---|---|---|---|---|---|---|
| Borlotti beans[1] | 0.5 | | | | | | | | | 100 | | | | | | |
| Cannellini beans[1] | 0.5 | | | | | | | | | 100 | | | | | | |
| Chickpeas[1] | 0.5 | | | | | | | | | 100 | | | | | | |
| Chickpeas flour[1] | 0.5 | | | | | | | | | 100 | | | | | | |
| Frozen peas[3] | 0.61 | | | | | | | | | 100 | | | | | | |
| Lentils[1] | 0.5 | | | | | | | | | 100 | | | | | | |
| Peas[1] | 0.5 | | | | | | | | | 100 | | | | | | |

Table S3: **Vegetables**

| Food item | CF (g $CO_2$/g) | White meat (%) | Red meat (%) | Cereals (%) | Sweets (%) | Fruit (%) | Fats (%) | Milk (%) | Cheese (%) | Legumes (%) | Olive oil (%) | Fish (%) | Vegetables (%) | Potatoes (%) | Eggs (%) | Sunflower oil (%) |
|---|---|---|---|---|---|---|---|---|---|---|---|---|---|---|---|---|
| Asparagus[1] | 0.7 | | | | | | | | | | | | 100 | | | |
| Cabbage[1] | 0.6 | | | | | | | | | | | | 100 | | | |
| Canned tomatoes[1] | 1.23 | | | | | | | | | | | | 100 | | | |
| Capers[1] | 0.91 | | | | | | | | | | | | 100 | | | |
| Carrots[1] | 0.14 | | | | | | | | | | | | 100 | | | |
| Cauliflower[1] | 0.6 | | | | | | | | | | | | 100 | | | |
| Celery[1] | 0.11 | | | | | | | | | | | | 100 | | | |
| Chard or swiss chard[1] | 0.1 | | | | | | | | | | | | 100 | | | |
| Condiriso[1] | 1.23 | | | | | | | | | | | | 100 | | | |
| Eggplant[1] | 1.35 | | | | | | | | | | | | 100 | | | |
| Fennel[1] | 0.14 | | | | | | | | | | | | 100 | | | |
| Fresh basil[1] | 0.91 | | | | | | | | | | | | 100 | | | |
| Frozen broccoli[3] | 0.814 | | | | | | | | | | | | 100 | | | |
| Frozen carrots[2] | 0.342 | | | | | | | | | | | | 100 | | | |
| Frozen cauliflower[3] | 0.773 | | | | | | | | | | | | 100 | | | |
| Frozen fennel[3] | 0.354 | | | | | | | | | | | | 100 | | | |
| Frozen potatoes[2] | 0.225 | | | | | | | | | | | | | 100 | | |
| Frozen spinach[2] | 0.277 | | | | | | | | | | | | 100 | | | |
| Frozen pumpkin[3] | 1.314 | | | | | | | | | | | | 100 | | | |
| Frozen zucchini[2] | 0.263 | | | | | | | | | | | | 100 | | | |
| Garlic[1] | 0.11 | | | | | | | | | | | | 100 | | | |
| Green beans[1] | 0.78 | | | | | | | | | | | | 100 | | | |
| Green salad[1] | 0.45 | | | | | | | | | | | | 100 | | | |
| Leaf vegetables[1] | 0.1 | | | | | | | | | | | | 100 | | | |
| Leeks[1] | 0.11 | | | | | | | | | | | | 100 | | | |
| Mushrooms[1] | 0.1 | | | | | | | | | | | | 100 | | | |
| Onions[1] | 0.11 | | | | | | | | | | | | 100 | | | |
| Origan[1] | 0.91 | | | | | | | | | | | | 100 | | | |
| Parsley[1] | 0.91 | | | | | | | | | | | | 100 | | | |
| Peppers[1] | 0.91 | | | | | | | | | | | | 100 | | | |
| Potatoes[1] | 0.13 | | | | | | | | | | | | | 100 | | |
| Pumpkin[1] | 1.1 | | | | | | | | | | | | 100 | | | |
| Red beets[1] | 0.11 | | | | | | | | | | | | 100 | | | |
| Rosemary[1] | 0.91 | | | | | | | | | | | | 100 | | | |
| Saffron[4] | 3205.13 | | | | | | | | | | | | 100 | | | |
| Sage[1] | 0.91 | | | | | | | | | | | | 100 | | | |
| Spinach[1] | 0.1 | | | | | | | | | | | | 100 | | | |
| Tomatoes[1] | 0.77 | | | | | | | | | | | | 100 | | | |
| Zucchini[1] | 1 | | | | | | | | | | | | 100 | | | |

Table S4: **Fruits**

| Food item | CF (g $CO_2$/g) | White meat (%) | Red meat (%) | Cereals (%) | Sweets (%) | Fruit (%) | Fats (%) | Milk (%) | Cheese (%) | Legumes (%) | Olive oil (%) | Fish (%) | Vegetables (%) | Potatoes (%) | Eggs (%) | Sunflower oil (%) |
|---|---|---|---|---|---|---|---|---|---|---|---|---|---|---|---|---|
| Apple[1, C] | 0.14 | | | | | 100 | | | | | | | | | | |
| Apricot[1, C] | 0.24 | | | | | 100 | | | | | | | | | | |
| Banana[1, C] | 1.35 | | | | | 100 | | | | | | | | | | |
| Black olives[1, C] | 0.52 | | | | | 100 | | | | | | | | | | |
| Grape[1, C] | 0.9 | | | | | 100 | | | | | | | | | | |
| Green olives[1, C] | 0.52 | | | | | 100 | | | | | | | | | | |
| Mandarin[1, C] | 0.36 | | | | | 100 | | | | | | | | | | |
| Orange[1, C] | 0.25 | | | | | 100 | | | | | | | | | | |
| Peach[1, C] | 0.24 | | | | | 100 | | | | | | | | | | |
| Pear[1, C] | 0.31 | | | | | 100 | | | | | | | | | | |
| Prune[1, C] | 0.24 | | | | | 100 | | | | | | | | | | |

Table S5: **Meat, meat products, and substitutes**

| Food item | CF (g $CO_2$/g) | White meat (%) | Red meat (%) | Cereals (%) | Sweets (%) | Fruit (%) | Fats (%) | Milk (%) | Cheese (%) | Legumes (%) | Olive oil (%) | Fish (%) | Vegetables (%) | Potatoes (%) | Eggs (%) | Sunflower oil (%) |
|---|---|---|---|---|---|---|---|---|---|---|---|---|---|---|---|---|
| Beef chuck[1] | 15.45 | | 100 | | | | | | | | | | | | | |
| Beef meatballs[1] | 15.45 | | 100 | | | | | | | | | | | | | |
| Chicken[1] | 1.88 | 100 | | | | | | | | | | | | | | |
| Chicken breast[1] | 1.88 | 100 | | | | | | | | | | | | | | |
| Chicken chops[1] | 1.88 | 100 | | | | | | | | | | | | | | |
| Chicken thigh[1] | 1.88 | 100 | | | | | | | | | | | | | | |
| Frozen chicken[5] | 3.65 | 100 | | | | | | | | | | | | | | |
| Cotechino[1] | 15.03 | | 100 | | | | | | | | | | | | | |
| Ground beef[1] | 15.45 | | 100 | | | | | | | | | | | | | |
| Ground chicken[1] | 1.88 | 100 | | | | | | | | | | | | | | |
| Ground turkey[1] | 1.88 | 100 | | | | | | | | | | | | | | |
| Ground veal[1] | 15.45 | | 100 | | | | | | | | | | | | | |
| Ham[1] | 15.03 | | 100 | | | | | | | | | | | | | |
| Lamb roll[1] | 13.85 | | 100 | | | | | | | | | | | | | |
| Mortadella[1] | 15.03 | | 100 | | | | | | | | | | | | | |
| Pork loin[1] | 3.65 | | 100 | | | | | | | | | | | | | |
| Pork sausage[1] | 15.03 | | 100 | | | | | | | | | | | | | |
| Roast gravy[4] | 4.41 | | 100 | | | | | | | | | | | | | |
| Salami[1] | 15.03 | | 100 | | | | | | | | | | | | | |
| Smoked beef[1] | 15.03 | | 100 | | | | | | | | | | | | | |
| Turkey[1] | 1.88 | 100 | | | | | | | | | | | | | | |
| Turkey breast[1] | 1.88 | 100 | | | | | | | | | | | | | | |
| Processed turkey meat[1] | 15.03 | 100 | | | | | | | | | | | | | | |
| Veal chuck[1] | 15.45 | | 100 | | | | | | | | | | | | | |
| Veal sirloin[1] | 15.45 | | 100 | | | | | | | | | | | | | |

Table S6: **Eggs and eggs products**

| Food item | CF (g $CO_2$/g) | White meat (%) | Red meat (%) | Cereals (%) | Sweets (%) | Fruit (%) | Fats (%) | Milk (%) | Cheese (%) | Legumes (%) | Olive oil (%) | Fish (%) | Vegetables (%) | Potatoes (%) | Eggs (%) | Sunflower oil (%) |
|---|---|---|---|---|---|---|---|---|---|---|---|---|---|---|---|---|
| Egg[1] | 2.7 | | | | | | | | | | | | | | 100 | |
| Hard-boiled eggs[1] | 2.7 | | | | | | | | | | | | | | 100 | |
| Pasteurized eggs[1] | 2.7 | | | | | | | | | | | | | | 100 | |

Table S7: **Fish and seafood**

| Food item | CF (g $CO_2$/g) | White meat (%) | Red meat (%) | Cereals (%) | Sweets (%) | Fruit (%) | Fats (%) | Milk (%) | Cheese (%) | Legumes (%) | Olive oil (%) | Fish (%) | Vegetables (%) | Potatoes (%) | Eggs (%) | Sunflower oil (%) |
|---|---|---|---|---|---|---|---|---|---|---|---|---|---|---|---|---|
| Blue shark fillet[1] | 2.27 | | | | | | | | | | | 100 | | | | |
| Cod cutlet[1] | 3.1 | | | | | | | | | | | 100 | | | | |
| Cod fillet[1] | 3.1 | | | | | | | | | | | 100 | | | | |
| Fish sticks[2] | 2.883 | | | | | | | | | | | 100 | | | | |
| Octopus[1] | 6.7 | | | | | | | | | | | 100 | | | | |
| Seafoods[1] | 6.7 | | | | | | | | | | | 100 | | | | |
| Tuna fish[1] | 2.27 | | | | | | | | | | | 100 | | | | |

Table S8: **Milk, milk products, and their substitutes**

| Food item | CF (g $CO_2$/g) | White meat (%) | Red meat (%) | Cereals (%) | Sweets (%) | Fruit (%) | Fats (%) | Milk (%) | Cheese (%) | Legumes (%) | Olive oil (%) | Fish (%) | Vegetables (%) | Potatoes (%) | Eggs (%) | Sunflower oil (%) |
|---|---|---|---|---|---|---|---|---|---|---|---|---|---|---|---|---|
| Cream[1] | 6 | | | | | | 100 | | | | | | | | | |
| Crescenza cheese[1] | 1.68 | | | | | | | | 100 | | | | | | | |
| Diced mozzarella cheese[1] | 10.14 | | | | | | | | 100 | | | | | | | |
| Edamer cheese[1] | 14 | | | | | | | | 100 | | | | | | | |
| Emmental cheese[1] | 14 | | | | | | | | 100 | | | | | | | |
| Fontal cheese[1] | 14 | | | | | | | | 100 | | | | | | | |
| Grated cheese[1] | 5.87 | | | | | | | | 100 | | | | | | | |
| Gorgonzola cheese[1] | 1.68 | | | | | | | | 100 | | | | | | | |
| Grana Padano cheese[1] | 4.6 | | | | | | | | 100 | | | | | | | |
| Mascarpone cheese[1] | 1.68 | | | | | | | | 100 | | | | | | | |
| Milk[1] | 1.31 | | | | | | | 100 | | | | | | | | |
| Mozzarella cheese[1] | 10.14 | | | | | | | | 100 | | | | | | | |
| Parmesan cheese[1] | 4.6 | | | | | | | | 100 | | | | | | | |
| Primosale cheese[1] | 1.68 | | | | | | | | 100 | | | | | | | |
| Ricotta cheese[1] | 2.52 | | | | | | | | 100 | | | | | | | |
| Robiola cheese[1] | 1.68 | | | | | | | | 100 | | | | | | | |
| Seasoned Tometta[1] | 4.6 | | | | | | | | 100 | | | | | | | |
| Spreadable fresh cheese[1] | 1.68 | | | | | | | | 100 | | | | | | | |
| Taleggio cheese[1] | 4.6 | | | | | | | | 100 | | | | | | | |
| Tomino[1] | 4.6 | | | | | | | | 100 | | | | | | | |
| Yogurt[1] | 1.5 | | | | | | | 100 | | | | | | | | |

Table S9: **Oil, fats spices, and sauces**

| Food item | CF (g $CO_2$/g) | White meat (%) | Red meat (%) | Cereals (%) | Sweets (%) | Fruit (%) | Fats (%) | Milk (%) | Cheese (%) | Legumes (%) | Olive oil (%) | Fish (%) | Vegetables (%) | Potatoes (%) | Eggs (%) | Sunflower oil (%) |
|---|---|---|---|---|---|---|---|---|---|---|---|---|---|---|---|---|
| Butter[1] | 3.36 | | | | | | 100 | | | | | | | | | |
| Herbs[1] | 0.91 | | | | | | | | | | | | 100 | | | |
| Mayonnaise[1] | 6 | | | | | | | | | | | | | | 20 | 80 |
| Olive oil[1] | 5.12 | | | | | | | | | | 100 | | | | | |
| Pepper[1] | 0.91 | | | | | | | | | | | | | | | |
| Pesto[3] | 2.72 | | | | | | | | | 33 | | | 34 | | | 33 |
| Sunflower oil[1] | 1.93 | | | | | | | | | | | | | | | 100 |
| Vegetable broth[2] | 0.591 | | | | | | | | | | | | 100 | | | |

Table S10: **Sweet products and substitutes**

| Food item | CF (g $CO_2$/g) | White meat (%) | Red meat (%) | Cereals (%) | Sweets (%) | Fruit (%) | Fats (%) | Milk (%) | Cheese (%) | Legumes (%) | Olive oil (%) | Fish (%) | Vegetables (%) | Potatoes (%) | Eggs (%) | Sunflower oil (%) |
|---|---|---|---|---|---|---|---|---|---|---|---|---|---|---|---|---|
| Biscuits[1] | 1.4 | | | | 100 | | | | | | | | | | | |
| Chocolate[1] | 4.2 | | | | 100 | | | | | | | | | | | |
| Ice-cream[1] | 4.14 | | | | 100 | | | | | | | | | | | |
| Jam[1] | 1.63 | | | | 100 | | | | | | | | | | | |
| Pudding[1] | 2.08 | | | | 100 | | | | | | | | | | | |
| Sugar[1] | 1.63 | | | | 100 | | | | | | | | | | | |
| Sweets[1] | 2.08 | | | | 100 | | | | | | | | | | | |
| Tarts[1] | 2.08 | | | | 100 | | | | | | | | | | | |

Table S11: **Non-alcoholic beverages**

| Food item | CF (g $CO_2$/g) | White meat (%) | Red meat (%) | Cereals (%) | Sweets (%) | Fruit (%) | Fats (%) | Milk (%) | Cheese (%) | Legumes (%) | Olive oil (%) | Fish (%) | Vegetables (%) | Potatoes (%) | Eggs (%) | Sunflower oil (%) |
|---|---|---|---|---|---|---|---|---|---|---|---|---|---|---|---|---|
| Decaffeinated coffee[1] | 1.43 | | | | | | | | | | | | | | | |
| Lemon juice[1] | 0.67 | | | | | | | | | | | | | | | |
| Tea[1] | 3.1 | | | | | | | | | | | | | | | |
| White vinegar[6] | 2.54 | | | | | | | | | | | | | | | |

Table S12: **Alcoholic beverages**

| Food item | CF (g $CO_2$/g) | White meat (%) | Red meat (%) | Cereals (%) | Sweets (%) | Fruit (%) | Fats (%) | Milk (%) | Cheese (%) | Legumes (%) | Olive oil (%) | Fish (%) | Vegetables (%) | Potatoes (%) | Eggs (%) | Sunflower oil (%) |
|---|---|---|---|---|---|---|---|---|---|---|---|---|---|---|---|---|
| Red wine[1] | 1.39 | | | | | | | | | | | | | | | |
| White wine[1] | 1.39 | | | | | | | | | | | | | | | |

Table S13: **Miscellaneous**

| Food item | CF (g $CO_2$/g) | White meat (%) | Red meat (%) | Cereals (%) | Sweets (%) | Fruit (%) | Fats (%) | Milk (%) | Cheese (%) | Legumes (%) | Olive oil (%) | Fish (%) | Vegetables (%) | Potatoes (%) | Eggs (%) | Sunflower oil (%) |
|---|---|---|---|---|---|---|---|---|---|---|---|---|---|---|---|---|
| Fresh fruit[1, C] | 0.234 | | | | | 100 | | | | | | | | | | |
| Fruit mousse[1, D] | 0.754 | | | | | 100 | | | | | | | | | | |
| Grain and legume soup[1, E] | 0.59 | | | 66 | | | | | | 34 | | | | | | |
| Mixed cereals[1, F] | 0.57 | | | 100 | | | | | | | | | | | | |
| Mixed legumes[1, G] | 0.5 | | | | | | | | | 100 | | | | | | |
| Vegetables tris[1, H] | 0.45 | | | | | | | | | | | | 100 | | | |

**1:** Ferrari M, Benvenuti L, Rossi L, et al. Could Dietary Goals and Climate Change Mitigation Be Achieved Through Optimized Diet? The Experience of Modeling the National Food Consumption Data in Italy. Front Nutr. 2020;7. doi:10.3389/fnut.2020.00048.
**2:** European Commission. Directorate General for Research and Innovation. Urban Food System Transformation in the Context of Food 2030: Current Practice & Outlook towards 2030. Publications Office; 2023. doi:10.2777/507125.
**3:** Wrobel-Jedrzejewska M, Polak E. Determination of carbon footprint in the processing of frozen vegetables using an online energy measurement system. Journal of Food Engineering. 2022;322:110974. doi:10.1016/j.jfoodeng.2022.110974.
**4:** Volanti M, Arfelli F, Neri E, et al. Environmental Impact of Meals: How Big Is the Carbon Footprint in the School Canteens? Foods. 2022;11(2):193. doi:10.3390/foods11020193.
**5:** Frederiksen R, Dalgaard R, Halberg N. LCA Food Database. Accessed December 14, 2023. https://www.lcafood.dk/.
**6:** Bartocci P, Fantozzi P, Fantozzi F. Environmental impact of Sagrantino and Grechetto grapes cultivation for wine and vinegar production in central Italy. Journal of Cleaner Production. 2017;140:569-580. doi:10.1016/j.jclepro.2016.04.090.

**A:** 18% of semolina, 4% of eggs, 2% of butter, 70% of milk and 6% of Parmesan cheese.
**B:** 36% of butter, 46% of flour and 18% of water.
**C:** The CF of fresh fruit was calculated as the mean value of the seasonal fruits indicated in the fruit section.
**D:** 50% of apple and 50% of banana.
**E:** 33% of lentils, 33% of spelt and 33% of barley.
**F:** 33% of spelt, 33% of barley and 33% of rice.
**G:** Mixed legumes: 50% of lentils, 50% of beans.
**H:** 33% of cauliflower, 33% of carrots and 33% of broccoli.
